# Vertical transmission of scrub typhus: a case report of congenital infection

**DOI:** 10.1080/22221751.2025.2542242

**Published:** 2025-07-29

**Authors:** Yonghan Luo, Zhenghui Liao, Xianyao Yang, Hechun Li, Jinyu Chi, Zhifang Cha, Jun Liu, Chuangang Ding, Yue Feng

**Affiliations:** a Faculty of Life Science and Technology, Kunming University of Science and Technology, Kunming, People’s Republic of China; b Second Department of Infectious Disease, Kunming Children's Hospital, Yunnan Key Specialty of Pediatric Infection (Training and Education Program)/Kunming Key Specialty of Pediatric Infection, Kunming, Yunnan, People’s Republic of China; c Pediatric Department, NO.1 people's hospital of Dali city, Dali, People’s Republic of China; d Clinical Laboratory, NO.1 people's hospital of Dali city, Dali, People’s Republic of China; e Imaging Department, NO.1 people's hospital of Dali city, Dali, People’s Republic of China; f Pediatric Department, The First Affiliated Hospital of Dali University, Dali, People’s Republic of China; g Yunnan Provincial Key Laboratory of Public Health and Biosafety, Kunming Medical University, Kunming, People’s Republic of China

**Keywords:** Congenital scrub typhus, vertical transmission, *Orientia tsutsugamushi*, systematic review, neonatal sepsis

## Abstract

Scrub typhus (ST), caused by Orientia tsutsugamushi, is typically transmitted through mite bites, but vertical transmission from mother to infant remains poorly understood. This case presents a 13-day-old female neonate diagnosed with congenital ST. The infant developed severe systemic involvement, including fever, jaundice, respiratory distress, and signs of hemophagocytic lymphohistiocytosis (HLH). Despite broad-spectrum antibiotic therapy, her condition worsened. PCR and serological tests confirmed the diagnosis of ST in both the neonate and the mother, with IgM positivity indicating congenital infection. Following treatment with doxycycline, the patient's condition improved. This case adds to the growing body of evidence supporting the vertical transmission of ST and highlights the need for increased awareness of congenital ST.

Scrub typhus (ST), also known as tsutsugamushi disease, is a disease caused by infection with *Orientia tsutsugamushi* [1]. The clinical manifestations typically include high fever, lymphadenopathy, rash, headache, myalgia, and arthralgia. Additionally, an eschar and ulceration may develop at the site of the mite bite, serving as a characteristic feature of the disease. In severe cases, ST can lead to multiple organ dysfunction syndrome (MODS), affecting the liver, kidneys, lungs, and brain, and may even result in coma or death [2]. The primary mode of transmission is through the bite of larval stage mites (“chiggers”), which facilitate horizontal transmission of the pathogen to humans. Research on the vertical transmission of ST – where the pathogen is passed from mother to infant – remains limited, and substantial evidence supporting a significant vertical transmission capability is lacking. Nevertheless, a few reports [3–10] have documented potential cases of congenital ST. Here, we presented a case of congenital ST, which may serve as evidence supporting the possibility of vertical transmission of the disease.

A 13-day-old female infant, G1P1, born at 38 + 6 weeks of gestation via spontaneous vaginal delivery, with a birth weight of 3.25 kg, presented with fever for three days prior to admission. The peak temperature was 39°C, accompanied by nasal congestion and tachypnea. There were no abnormalities in the birth history. The mother, a 21-year-old primigravida, had undergone routine prenatal check-ups without reports of fever, premature rupture of membranes, or gestational diabetes. The diagnosis and management of congenital ST in this case are presented in S1 and Figure 1.

**Figure 1.**
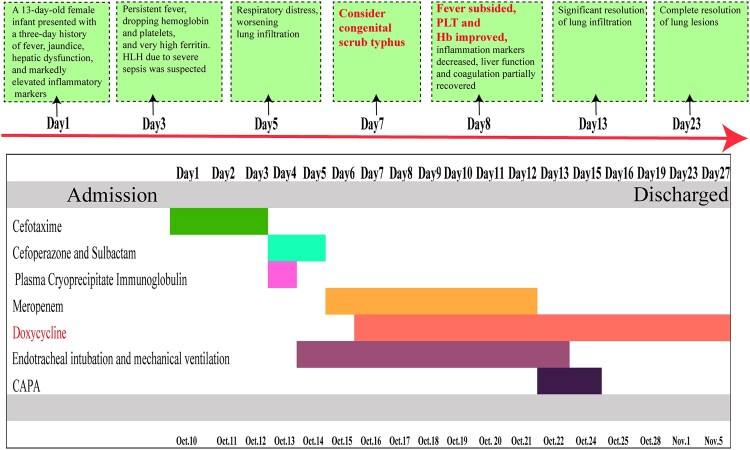
Diagnosis, treatment, and the progression of disease.

Upon admission, the infant had a body temperature of 39.4°C, heart rate of 190 bpm, respiratory rate of 58 breaths/min, and blood pressure of 79/34 mmHg. Her weight was 3.3 kg. Moderate jaundice of the skin and mucosa, along with scleral icterus, was observed. No signs of intercostal retractions were noted, and the cardiovascular, pulmonary, and abdominal examinations revealed no significant abnormalities.

The laboratory evaluation revealed a WBC count of 5.6 × 10^9^/L, with neutrophils at 2.96 × 10^9^/L and lymphocytes at 1.92 × 10^9^/L. The patient presented with a hemoglobin concentration of 147.0 g/L and a platelet count of 64 × 10^9^/L. Inflammatory markers were markedly elevated, with a CRP level of 63.19 mg/L and a procalcitonin concentration of 3.560 ng/mL. Liver function tests indicated significant hyperbilirubinemia, with a total bilirubin level of 205.4 µmol/L, direct bilirubin at 40.3 µmol/L, and indirect bilirubin at 165.1 µmol/L. Hypoalbuminemia (35.2 g/L) was observed alongside elevated ALT (92 U/L), AST (312 U/L), and LDH (1289 U/L). Coagulation studies revealed reduced fibrinogen levels at 1.74 g/L. Cerebrospinal fluid analysis demonstrated a WBC count of 2 × 10^6^/L, chloride concentration of 124 mmol/L, protein level of 0.79 g/L, and glucose concentration of 3.11 mmol/L.

Upon admission, the febrile episode was initially suspected to be caused by a bacterial infection, and cefotaxime was administered for antimicrobial therapy (Day 1–Day 3). Daily monitoring of complete blood count (CBC) and liver and renal function was conducted. By Day 3, the patient exhibited persistent recurrent fever, with a progressive decline in hemoglobin (Hb) and platelet count (PLT): Hb decreased from 147 to 103 g/L, and PLT from 64 × 10^9^/L to 47 × 10^9^/L. Additionally, fibrinogen levels declined from 1.74 to 0.8 g/L, C-reactive protein (CRP) increased from 63.19 to 83.9 mg/L, and lactate dehydrogenase (LDH) levels showed a progressive rise from 1289 to 2154 U/L. Alanine aminotransferase (ALT) and aspartate aminotransferase (AST) levels also demonstrated a marked increase, with ALT rising from 92 to 236 U/L and AST from 312 to 644 U/L. Ferritin levels were significantly elevated (>2000 ng/mL), raising suspicion of hemophagocytic lymphohistiocytosis (HLH) secondary to severe sepsis. Consequently, antibiotic therapy was escalated to cefoperazone-sulbactam (Day 4–Day 5) and meropenem (Day 6–Day 12).

The patient subsequently developed pronounced respiratory distress, characterized by significant tachypnea and intercostal retractions. A follow-up chest X-ray revealed progression of bilateral pulmonary infiltrates, necessitating endotracheal intubation and mechanical ventilation (Day 5–Day 12). Supportive measures included administration of plasma, cryoprecipitate for coagulation factor supplementation, and intravenous immunoglobulin (IVIG) for immune modulation.

On Day 7, a detailed maternal history revealed that the mother had experienced a one-day febrile episode prenatally, accompanied by an eschar (0.5 × 0.5 cm) on the medial aspect of her right thigh (Figure 2(A)). She had been diagnosed with ST postpartum and received unspecified anti-rickettsial therapy, with fever resolution by the fifth postpartum day. Blood samples from both the infant and the mother underwent PCR analysis for the detection of Rickettsia. Qualitative qPCR targeting the groEL gene produced a positive result, demonstrating a consistent genotype. A cycle threshold (Ct) value below 38 was considered indicative of positivity, and the infant's sample yielded a Ct value of 30.44. Subsequently, nested PCR was employed to amplify the target fragment, followed by sequencing of the TSA56 gene. Alignment of the resulting sequence further confirmed the presence of Orientia tsutsugamushi (Karp-C subtype), as illustrated in the phylogenetic tree shown in Figure 2(B). Moreover, the maternal blood sample tested positive for OXK in the Weil-Felix test with an antibody titer of ≥1:320, while the infant’s blood sample tested positive for OXK with a titer of 1:160 (a titer >1:80 is considered positive), which further confirmed the diagnosis of scrub typhus. In addition, the sera from both the mother and the neonate were analysed using the InBIOS Scrub Typhus ELISA, and both exhibited concurrent IgG and IgM positivity: the mother showed IgG OD = 1.507 (cutoff ≥0.30) and IgM OD = 3.967 (cutoff ≥0.28) at a 1:100 dilution, while the neonate exhibited IgG OD = 3.148 (Positive) and IgM OD = 2.700 (Positive) at the same dilution. Critically, the neonatal IgM positivity indicates the production of new antibodies, providing serological proof of congenital infection. Doxycycline (2.2 mg/kg/dose, BID) was introduced for antimicrobial therapy. By the second day of doxycycline administration, the patient became afebrile, with improvements in PLT and Hb levels, a reduction in inflammatory markers, and partial recovery of liver function and coagulation parameters.

**Figure 2.**
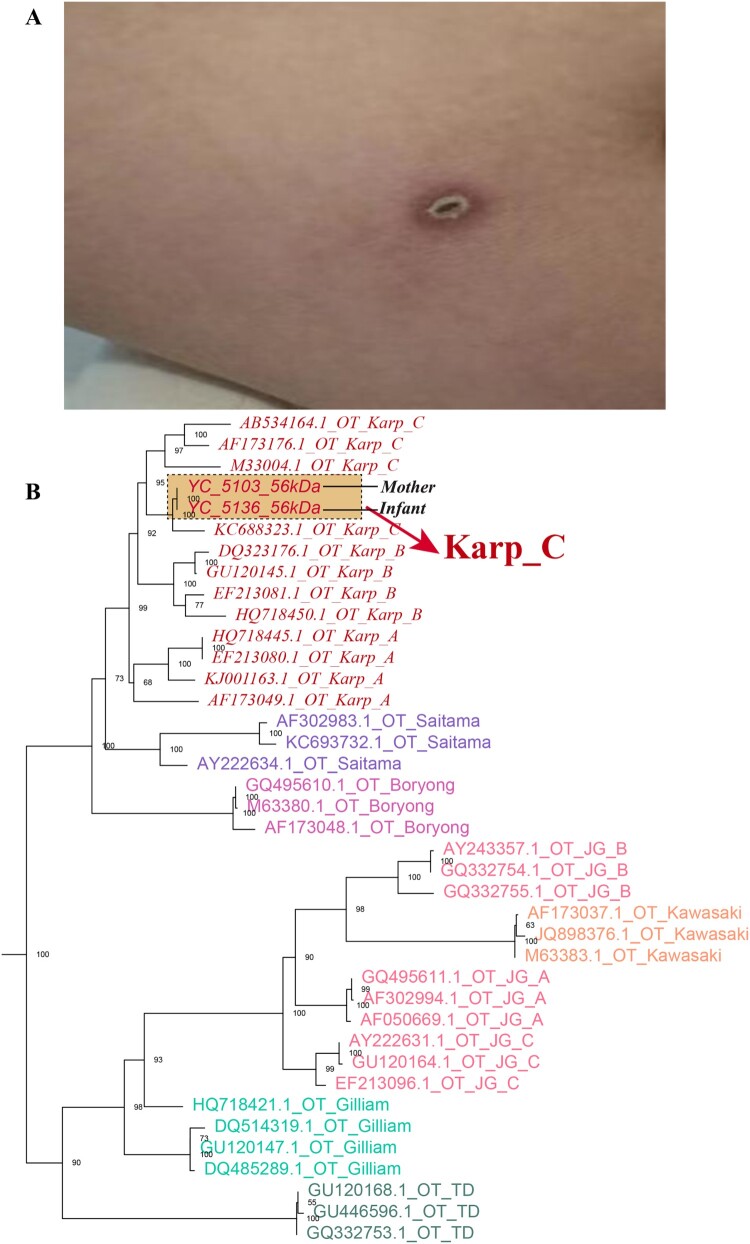
**A.** The patient’s mother, a 21-year-old woman, experienced fever one day before delivery, with a 0.5 × 0.5 cm eschar observed on the medial aspect of her right thigh. **B.** Maximum likelihood phylogenetic tree of Orientia tsutsugamushi based on the 56 kDa type-specific antigen gene sequences.

By Day 13, follow-up chest radiography showed significant resolution of pulmonary infiltrates, with further normalization of inflammatory markers, liver function, and coagulation indices. Mechanical ventilation was discontinued, and continuous positive airway pressure (CPAP) support was provided (Day 13–Day 16), allowing for the cessation of meropenem therapy. After two weeks of doxycycline treatment, follow-up chest imaging confirmed near-complete resolution of pulmonary lesions. The patient remained afebrile, demonstrated adequate oral intake, and was subsequently discharged.

## Discussion

Congenital ST is a rare condition, with a limited number of reported cases in the literature. We conducted a search through databases such as PubMed and Web of Science, identifying 10 case reports [3–10], in addition to the case we present, bringing the total number of cases to 11. This case provides further evidence supporting the possibility of vertical transmission of *Orientia tsutsugamushi* from mother to infant.

*Orientia tsutsugamushi* is primarily transmitted through the bite of infected chiggers, but the vertical transmission in humans remains poorly understood. Some case reports, including ours, suggest the potential for in utero transmission. The earliest signs in neonates appear within the first few days after birth, indicating that unexplained fever at this stage could strongly suggest an intrauterine infection rather than environmental exposure. In our case, despite broad-spectrum antibiotic treatment, the neonate's condition progressively worsened within 10 days of birth, consistent with previously reported [3,4,6] neonatal cases. Eschar is a hallmark feature for identifying ST [11], but it is predominantly associated with horizontally transmitted (mite-acquired) infection. In contrast, our vertically infected neonates lacked the distinctive eschar, even though it is not always present in children with acquired ST [12]. Therefore, diagnosing neonatal ST requires a detailed inquiry into the mother's pregnancy history. Both our case and a case reported by Liang et al. describe the mother having fever with eschar during pregnancy, indirectly supporting the hypothesis of placental transmission.

The clinical presentation of congenital ST is highly variable, ranging from mild febrile illness to life-threatening multi-organ dysfunction. In our case, the neonate exhibited severe systemic involvement, persistent fever, and widespread inflammation. A review of neonatal ST indicates that all affected neonates presented with fever, and despite initial broad-spectrum antibiotic treatment, our patient continued to experience high fever, similar to other neonatal cases [3–10]. Laboratory markers, like those observed in ST across all age groups, revealed progressive declines in hemoglobin and platelets. We also noted signs of hemophagocytic lymphohistiocytosis (HLH), such as a significant decrease in fibrinogen levels and an increase in ferritin, suggesting that *O. tsutsugamushi* may trigger excessive immune activation in neonates [13]. However, unlike HLH caused by other infectious diseases, such as Epstein–Barr virus (EBV), the hemophagocytic markers in our patient rapidly normalized with effective anti-infective treatment, highlighting a distinct mechanism that warrants further investigation.

Due to the lack of specific clinical features and the rarity of reported cases, the diagnosis of congenital ST remains challenging. As seen in our patient and previous reports, delayed recognition of ST typically results in prolonged empirical antibiotic therapy without clinical improvement. Currently, serological tests and PCR-based molecular diagnostics remain the primary laboratory diagnostic methods for*. tsutsugamushi* [14]. PCR can detect pathogen DNA during the acute phase, providing an earlier diagnosis compared to serological methods, particularly when neonates have not yet developed sufficient antibodies. However, PCR requires amplification of specific genes, and false-negative results may occur if the detection window is missed or if bacteremia is at a low level. In recent years, Next-Generation Sequencing (NGS) has opened new opportunities for accurate diagnosis of ST. Unlike traditional PCR, which requires known target genes, NGS can directly detect and identify *O. tsutsugamushi* from blood, cerebrospinal fluid, or other sterile body fluids, even in cases with low pathogen load or atypical clinical presentations [15]. Therefore, NGS could serve as an important supplementary diagnostic tool, providing critical support for early identification and precise treatment.

Regarding neonatal treatment, a review of approximately ten cases reveals significant variability in treatment regimens, including the use of chloramphenicol, azithromycin, and doxycycline. Doxycycline remains the most widely used treatment option. In our case, initiating doxycycline led to rapid defervescence, hematologic recovery, and resolution of systemic inflammation. Despite prior concerns about tooth discolouration, the increase in pediatric cases of mycoplasma pneumonia resistant to macrolides in recent years has led to doxycycline becoming a preferred choice after macrolide treatment failure in children under eight years [16,17], indirectly confirming its safety in young children. Another critical point is the importance of early effective treatment of rickettsial infections, as delayed treatment has been associated with increased morbidity and mortality in both neonates and adults.

## Conclusion

Our case contributes to the growing body of evidence supporting the vertical transmission of *O. tsutsugamushi* and highlights the need for increased awareness of congenital ST in neonates with unexplained sepsis, especially in endemic regions. Further research is warranted to elucidate the mechanisms of vertical transmission and to establish diagnostic and therapeutic guidelines for perinatal ST infection.

## Supplementary Material

S1.docx
