# Supplementary material for: Vertical transmission of scrub typhus: a case report of congenital infection
Source: Emerg Microbes Infect. 2025 Jul 29;14(1):2542242. doi: 10.1080/22221751.2025.2542242 (PMC12337731; doi:10.1080/22221751.2025.2542242)
Supplement: S1.docx [file TEMI_A_2542242_SM5371.docx]

**S1.** Diagnosis and treatment of congenital ST in this case.

| **Date** | **10.10** | **10.11** | **10.12** | **10.13** | **10.14** | **10.15** | **10.16** | **10.17** | **10.18** | **10.19** | **10. 20** | **10.21** | **10.22** | **10.24** | **10.25** | **10.28** | **11.1** | **11.5** | **Normal range** |
| --- | --- | --- | --- | --- | --- | --- | --- | --- | --- | --- | --- | --- | --- | --- | --- | --- | --- | --- | --- |
| **White Blood Cell, 10⁹/L** | 5.6 | 5 | 6.3 | 7.9 | 10.5 | 13.7 | 13.2 | 27.5 | 14.5 | 8.7 | 8 |  |  | 7 |  |  | 8.8 |  | 4--10 |
| **Neutrophil Percentage, %** | 53.3 | 70.8 | 53.9 | 55.2 | 52.7 | 42 | 54.1 | 43.3 | 45.6 | 42 | 17.1 |  |  | 18.2 |  |  | 18.4 |  | 50-70 |
| **Lymphocyte Percentage, %** | 34.4 | 22.2 | 31.2 | 29.1 | 39 | 45 | 21.4 | 21.4 | 22.2 | 34.9 | 49.6 |  |  | 55.5 |  |  | 62.7 |  | 20-40 |
| **Hemoglobin, g/L** | 147 | 126 | 117 | 103 | 97 | 86 | 78 | 124 | 117 | 119 | 111 |  |  | 99 |  |  | 97 |  | 110-160 |
| **Platelet Count, 10⁹/L** | 64 | 62 | 47 | 47 | 33 | 43 | 51 | 51 | 54 | 64 | 102 |  |  | 337 |  |  | 316 |  | 100-300 |
| **Total Bilirubin, µmol/L** | 205.4 | 212.6 | 187.1 | 153.9 | 149.2 | 136.2 | 99.9 | 106.4 | 102.1 |  | 110.1 |  |  | 155.6 |  | 167.5 | 134.7 | 109.9 | 5.1-19 |
| **Direct Bilirubin, µmol/L** | 40.3 | 61.7 | 89.5 | 97.4 | 101.4 | 87.3 | 61.2 | 66 | 62.1 |  | 73.1 |  |  | 109.1 |  | 117.3 | 93.6 | 74.6 | 1.7-6.8 |
| **Indirect Bilirubin, µmol/L** | 165.1 | 150.9 | 97.6 | 56.5 | 47.8 | 48.9 | 38.7 | 40.4 | 40 |  | 37 |  |  | 46.5 |  | 50.2 | 41.1 | 35.3 | 0-13.7 |
| **Alanine Aminotransferase, U/L** | 92 | 105 | 176 | 236 | 340 | 421 | 378 | 375 | 298 |  | 197 |  |  | 233 |  | 287 | 192 | 131 | 0-31 |
| **Aspartate Aminotransferase, U/L** | 312 | 380 | 648 | 644 | 825 | 823 | 554 | 408 | 249 |  | 199 |  |  | 346 |  | 420 | 257 | 162 | 0-31 |
| **Lactate Dehydrogenase, U/L** | 1289 | 1517 | 2154 | 1871 | 1862 | 1677 | 1.92 | 841 | 612 |  | 508 |  |  |  |  |  |  |  | 135-124 |
| **C-Reactive Protein, mg/L** | 63.19 | 91.8 | 111.3 | 85.4 | 83.9 | 83.2 | 35.5 | 36.5 | 14.5 |  | 7.9 |  |  | 4.45 |  | 3.18 |  |  | 0-5 |
| **Procalcitonin, ng/mL** | 3.56 | 4.75 | 2.57 | 1.31 | 1.52 | 1.33 | 1.02 | 0.59 | 0.51 |  | 0.41 |  |  |  |  |  |  |  | 0-0.05 |
| **Fibrinogen, g/L** |  | 1.74 | 0.79 | 0.71 | 0.8 | 0.84 | 1.13 | 0.94 | 0.75 |  | 1.47 |  |  |  |  | 2.8 |  |  | 2--4 |
| **D-Dimer, μg/mL** |  | 57.94 | 62.06 | 31.67 | 14.62 | 25.78 | 51.98 | 65.97 | 18.86 |  | 4.69 |  |  |  |  |  |  |  | 0-1 |
| **Ferritin, ng/mL** |  |  |  |  | ＞2000 |  |  |  |  |  | ＞2000 |  |  |  |  |  |  |  |  |
| **Cerebrospinal Fluid** |  |  |  |  |  |  |  |  |  |  |  |  |  |  |  |  |  |  |  |
| **Leukocyte Count, 10⁶/L** |  | 2 |  |  |  | 9 |  |  |  | 0 |  | 0 |  |  | 8 |  | 2 | 1 |  |
| **Total Protein, g/L** |  | 791 |  |  |  | 3828 |  |  |  | 2362 |  | 1342 |  |  | 896 |  | 939 | 770 | 0-500 |
| **Chloride, mmol/L** |  | 124 |  |  |  | 106 |  |  |  | 118 |  | 124 |  |  | 128 |  | 126 | 124 | 120-132 |
| **Glucose, mmol/L** |  | 3.11 |  |  |  | 1.38 |  |  |  | 1.7 |  | 2.06 |  |  | 2.06 |  | 2.01 | 2.08 | 2.5--4.5 |
